# Effects of Different LVEF Assessed by Echocardiography and CMR on the Diagnosis and Therapeutic Decisions of Cardiovascular Diseases

**DOI:** 10.3389/fphys.2020.00679

**Published:** 2020-06-16

**Authors:** Lei Zhao, Aijia Lu, Jie Tian, Jie Huang, Xiaohai Ma

**Affiliations:** ^1^Department of Radiology, Beijing Anzhen Hospital, Capital Medical University, Beijing, China; ^2^Department of Radiology, Michigan State University, East Lansing, MI, United States; ^3^Department of Interventional Therapy, Beijing Anzhen Hospital, Capital Medical University, Beijing, China

**Keywords:** cardiac magnetic resonance, left ventricular ejection fraction, echocardiography, diagnosis, imaging

## Abstract

**Aims:**

The impact of different left ventricular ejection fraction (LVEF) assessed by echocardiography (EC) and cardiac magnetic resonance (CMR) on clinical diagnosis and management that could be critical in clinical practice remains unclear. This study investigated this impact for patients who underwent both exams in a real-world clinical practice.

**Methods:**

500 patients who underwent CMR and two-dimensional EC were retrospectively included in present study. EC-measured LVEF and CMR-measured LVEF were compared. A 50% cut-off of LVEF was chosen to assess the effect of the difference between these two modalities on disease diagnosis, and a 35% cut-off was chosen for disease management, respectively. For those patients who received device therapy or coronary artery bypass grafting (CABG), the study compared the LVEF between EC and CMR with the current guideline for therapy recommendation.

**Results:**

EC-LVEF and CMR-LVEF were positively correlated, but EC-LVEF was significantly larger than CMR-LVEF (*P* < 0.001). Three patient groups were examined: (I) CMR-LVEF ≥ 50%, (II) 35% < CMR-LVEF < 50%, and (III) CMR-LVEF ≤ 35%. Overall, 139 of 500 patients showed inconsistent measures. There were more inconsistent measures between the two modalities in group III than group I (41.6% for group III vs. 4.1% for group I). In patients who received device therapy or CABG, 97.6% of the CMR-measured LVEF were consistent with the guideline, but only 61.0% consistent EC-measured LVEF.

**Conclusion:**

For patients with lower LVEF and planning to receive device therapy or cardiac surgery, it should be cautious to applying the recommended cut-off values to CMR-measured LVEF because its inconsistency with EC-measured LVEF.

## Introduction

Quantitative assessment of cardiac function is important for diagnosis and management of cardiovascular diseases. As the most commonly used index of cardiac function, left ventricular ejection fraction (LVEF) is extensively cited in the guidelines as one work-up indication for diagnosis and treatment of heart failure, arrhythmias, valvular heart disease, acute myocardial infarction, and non-ischemic heart disease, etc. The guidelines provide a LVEF cut-off value of 50% to diagnose heart failure with preserved EF or not (Ponikowski et al., 2016). For the treatment planning, the guidelines provide a LVEF cut-off value of 35% to determine revascularization for coronary artery disease and device therapy for arrhythmias (European Society of Cardiology [ESC] et al., 2013; Neumann et al., 2019). Cardiac magnetic resonance (CMR) is the gold standard for LVEF quantification (Ponikowski et al., 2016). As an easy assessable method, echocardiography (EC) is most commonly used to assess LVEF clinically. The two-dimensional (2D) EC, which is a simple and most frequently used EC method, may be less accurate under the condition of LV chamber measured in inappropriate imaging planes due to discordant ventricular function and asymmetric LV dilation (Thomas et al., 2012). Although the CMR-measured LVEF and EC-measured LVEF showed good correlation in both normal and diseased cohorts (Thomas et al., 2012; Wood et al., 2014), it remains unclear the effect of their different LVEF on clinical diagnosis and management that could be critical in clinical practice. This study investigated this effect for patients who underwent both CMR and EC in a real world clinical practice.

## Methods

We retrospectively examined 500 patients from January to May of 2018, and each patient underwent CMR (MAGNETOM Verio, Siemens Healthcare, Germany) and EC (Vivid E9, GE Health Medical, United States) with time interval ≤1 day. All CMR examinations were performed using a 3T MR system with a 32-channel cardiac coil. Steady-state free-precession cine images were obtained during repeated breath-holds in two long axes (horizontal and vertical) and in a stack of short axes covering the LV. Imaging parameters were: repetition time (TR) = 3.1 ms, echo time (TE) = 1.3 ms, asymmetric echo with factor 0.29, flip angle (FA) = 45°, field of view (FOV) = 276 × 340 mm^2^, matrix = 156 × 192, slice thickness = 6 mm, receiver bandwidth (BW) = 704 Hz/px, parallel imaging using GRAPPA reconstruction (*R* = 2), 30 cardiac phases. One experienced doctor with specialty in EC performed all the EC exams and one experienced doctor with specialty in CMR analyzed all the CMR data, respectively. For each individual, EC-LVEF was measured with unenhanced 2D echocardiography (modified Simpson apical view biplane method), and CMR-LVEF was measured with steady-state free procession short-axis cine CMR (Simpson disk summation method, papillary muscles were excluded when delineating endocardial borders). CMR-measured LV end-diastolic volume (LVEDV) was also recorded for each patient to perform the comparisons. After starting this retrospective study, the CMR-LVEF analysis was repeated by the same reader to assess the reproducibility. The study conducted three comparisons. (1) The correlations among these three measures were analyzed first to test their relationships. Then, EC-LVEF was compared with CMR-LVEF using paired *t*-test and intraclass correlation coefficients (ICCs) with 95% confidence intervals. The Bland-Altman analysis was used to examine whether EC over- or under-estimated LVEF compared to CMR. (2) A 50% cut-off CMR-LVEF was chosen to assess the effect of the difference between EC and CMR on disease diagnosis, and a 35% cut-off was chosen for disease management, respectively (European Society of Cardiology [ESC] et al., 2013; Ponikowski et al., 2016; Neumann et al., 2019). For each individual, the two measures were consistent with each other if both measures were larger or smaller than the cut-off value; otherwise, they were inconsistent. With these consistent and inconsistent measures, EC-LVEF was compared with CMR-LVEF to assess the effect of the difference between these two measures on disease diagnosis and management. (3) For those patients who received device therapy or coronary artery bypass grafting (CABG), the consistency of the measured LVEF was compared between EC and CMR with the current guideline for therapy recommendation, i.e., LVEF ≤ 35% for both device therapy for pacing and CABG in coronary artery disease with LV dysfunction (European Society of Cardiology [ESC] et al., 2013; Neumann et al., 2019).

## Results

**FIGURE 1:**
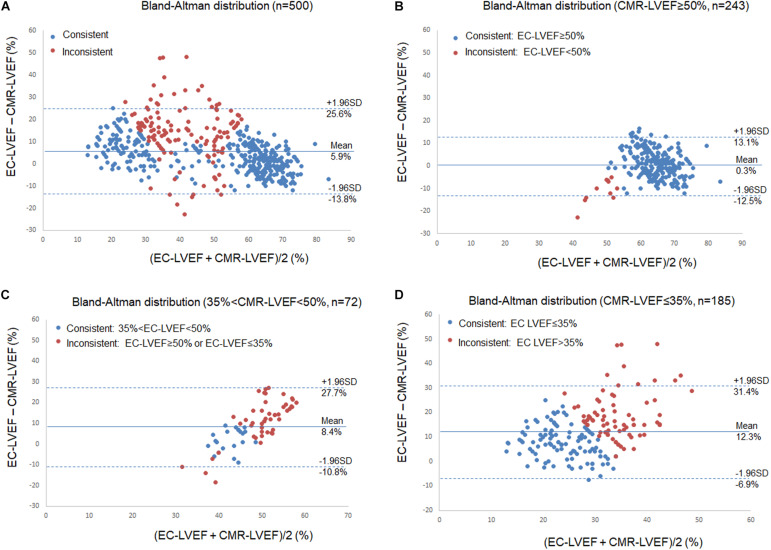
Bland-Altman Plot for Left Ventricular Ejection Fraction (LVEF). Comparisons of echocardiography (EC) LVEF with cardiac magnetic resonance (CMR) LVEF. Blue dots indicate those patients their LVEF were measured consistently between EC and CMR, and red dots indicated those with inconsistent measures. **(A)** all patients, 27.8% patients had inconsistent measures (139/500); **(B)** 243 patients with CMR-LVEF ≥ 50%, 4.5% patients (n = 11) had inconsistent measures; **(C)** 72 patients with 35% < CMR-LVEF < 50%, 70.8% patients (n = 51) had inconsistent measures; and **(D)** 185 patients with CMR-LVEF ≤ 35%, 41.6% patients (n = 77) had inconsistent measures.

In these 500 patients (343 men, age 49 ± 15 years), 121 had coronary artery disease, 73 had hypertrophic cardiomyopathy, 7 had cardiac amyloidosis, 3 had cardiac sarcoidosis, 144 heart failure, 78 arrhythmias, 30 valvular heart disease, 13 myocarditis, and 31 arterial hypertension. The CMR-LVEF intraobserver variability was 1.2 ± 8.8% for all cases, 1.6 ± 13.6% for cases with CMR-LVEF ≤ 35%, 2.0 ± 6.1% for CMR-LVEF between 35 and 50%, and 0.7 ± 3.0% for cases with CMR-LVEF ≥ 50%. EC-LVEF positively correlated with CMR-LVEF, and both negatively correlated with LVEDV (EC-LVEF vs. CMR-LVEF: *R* = 0.871, EC-LVEF vs. LVEDV: *R* = -0.613, and CMR-LVEF vs. LVEDV: *R* = -0.660, max *P* < 0.003 with Bonferroni correction), showing that the larger the LVEDV, the smaller the LVEF, regardless of EC and CMR. ICCs with 95% confidence intervals between the CMR- and EC-LVEF is 0.808 (95% CI: 0.626∼0.887). EC-LVEF was significantly larger than CMR-LVEF (*P* < 0.001, Table 1), and the Bland-Altman analysis showed that EC overestimated LVEF compared to CMR (Figure 1A).

**TABLE 1:** Comparison of LVEF between CMR and EC.

**Patients category (n)**	**CMR-LVEF (%)**	**EC-LVEF (%)**	**Correlation (*r*-, *P*-value)**	**EC-LVEF – CMR- LVEF (%) (95% CI)**	***P*-value**	**LVEDV (ml)**
All patients (*n* = 500)	45.4 ± 20.2	51.4 ± 16.1	0.871, <0.001	5.9 ± 10.0 (5.0%∼6.8%)	<0.001	139 ± 86
CMR-LVEF ≥ 50% (*n* = 243)	63.7 ± 7.0	64.0 ± 6.9	0.561, <0.001	0.3 ± 6.5% (-0.5%∼1.1%)	0.444	92 ± 32
35% < CMR-LVEF < 50% (*n* = 72)	43.8 ± 3.9	52.3 ± 10.1	0.263, 0.026	8.4 ± 9.8% (6.1%∼10.7%)	<0.001	154 ± 72*
CMR-LVEF ≤ 35% (*n* = 185)	22.1 ± 7.1	34.4 ± 10.1	0.399, <0.001	12.3 ± 9.8% (10.9%∼13.7%)	<0.001	213 ± 96*
Patients received device therapy or CABG (*n* = 41)	25.4 ± 13.7	37.0 ± 10.9	0.782, <0.001	11.6 ± 8.5% (8.9%∼14.3%)	<0.001	195 ± 82*

Data are presented as mean ± SD, otherwise indicated. *For each group indicated with *, its LVEDV was compared to that of the group CMR-LVEF ≥ 50% (t-test, max P < 0.001). LVEF, left ventricular ejection fraction; CMR, cardiac magnetic resonance; EC, echocardiography; LVEDV, left ventricular end-diastolic volume; CI, confidence interval; CABG, coronary artery bypass grafting.

To assess the potential effect of this EC-overestimated LVEF on disease diagnosis and management, CMR-LVEF was used to divide the patients into three groups: (I) those patients with CMR-LVEF ≥ 50%, (II) those with 35% < CMR-LVEF < 50%, and (III) those with CMR-LVEF ≤ 35% (Table 1). Overall, 139 of 500 patients (27.8%) showed inconsistent measures (Figure 1A). For group I (*n* = 243), only 10 patients (4.1%) showed inconsistent measures, and the mean of EC-LVEF was almost identical to that of CMR-LVEF (Table 1 and Figure 1B), demonstrating a similar accuracy of EC vs. CMR on the diagnosis for this group patients. For group II (*n* = 72), 51 patients (70.8%) showed inconsistent measures, and the mean of EC-LVEF was significantly larger than that of CMR-LVEF (*t*-test, *P* < 0.001) (Table 1 and Figure 1C), implying a potentially significant effect of this EC-overestimated LVEF on the disease diagnosis and management, and this effect may not be negligible for group II patients. For group III (*n* = 185), 77 patients (41.6%) showed inconsistent measures, and the mean of EC-LVEF showed a similar behavior to that of group II (Table 1 and Figure 1D), indicating the same potential effect of this EC-overestimated LVEF on the disease management. This effect may be more substantial for group III compared to group II (Table 1 and Figures 1C,D). Comparing to group I, LVEDV was significantly larger for group II (*t*-test, *P* < 0.001) and even more larger for group III (*t*-test, *P* < 0.001) (Table 1), consistent with the inverse relationship between LVEDV and LVEF.

To further assess the effect of this EC-overestimated LVEF on therapeutic decisions, we used the current guideline of LVEF ≤ 35% as the cut-off for both device therapy and CABG. A total of 30 patients who received device therapy (16 CRT/CRT-D and 14 implantable cardioverter defibrillator) and 11 patients who received CABG were identified. For these 41 patients, EC significantly overestimated LVEF compared to CMR (Pair *t*-test, *P* < 0.001, Table 1). Overall, 24 of 41 patients (58.5%) showed consistent LVEF between EC and CMR. A total of 40 patients (97.6%) with CMR-measured LVEF were consistent with the guideline. In contrast, only 25 patients (61.0%) with EC-measured LVEF were consistent with the guideline. This substantially higher consistent rate of 97.6% of the CMR-measured LVEF with the guideline demonstrates a superior reliability of CMR for the disease treatment in the condition of lower LVEF.

## Discussion

This study found that 2D EC overestimates LVEF compared to CMR and the degree of this overestimation is LVEF dependent. When LVEF is larger than 50%, EC-assessed LVEF is similar as CMR. When LVEF is less than 50%, particularly < 35%, however, EC significantly overestimates LVEF compared to CMR. Considering that the LVEF cut-off values in the guidelines are based on clinical trials with EC-assessed LVEF, it should be cautious to applying these cut-off values to CMR-assessed LVEF in decision making for cardiovascular disease diagnosis and management in the condition of lower LVEF because of the inconsistency between EC- and CMR-assessed LVEF, though CMR is the gold standard for LVEF quantification.

In the literature inconsistent results are reported about the LVEF measurement compared between EC and CMR. Simpson et al. (2018) report that EC underestimates LVEF compared to CMR, while others suggest that EC mildly overestimates LVEF compared to CMR (Wood et al., 2014). LVEF measurement can be affected by many factors such as LV remodeling. EC biplane and single plane methods rely on geometric assumptions. Geometric abnormalities of the left ventricle may also contribute to the inconsistency between these two modalities. LVEDV and end-systolic volume measured with EC methods are smaller and show greater variability than those derived from CMR (Wood et al., 2014). The impact of these factors may explain the observed discordance of LVEF measurement, and further studies with full source data are needed to understand this disagreement between the two modalities.

2D EC is easily accessible and widely used in clinical practice. However, as LVEF is inversely related with LVEDV, a reduced LVEF is associated with an enlarged LVEDV (Table 1), and it might be difficult to accurately assess the LVEF with clinical commonly used biplane echocardiography, particularly under the condition of pathological asymmetric LV remodeling. 3-dimensional (3D) EC may improve the accuracy of assessing LVEF because of the absence of geometric assumptions pertaining to LV contour (Benameur et al., 2019; Rodriguez-Mañero et al., 2019). Routine use of 3D (contrast enhanced) EC may allow more accurate cardiac function (Thomas et al., 2012), but more evidence are needed to provide LVEF cut-offs for clinic because the recommended LVEF cut-offs in the guidelines are based on clinical trials in which many used 2D EC to assess LVEF.

For this retrospective study, the intraobserver variability of the CMR-LVEF is small with an overall variability of 1.2 ± 8.8% for all cases. As we could not assess the intraobserver variability of the EC-LVEF and lack the EC-measured LVEDV (i.e., LVEDV was not recorded on the EC exam report), we cannot compare them between the two modalities. In addition, as singe reader performed the CMR analysis, we could not assess the interobserver variability. However, previous studies report that the reproducibility of CMR is better than the non-contrast 2D EC, and LVEDV measured by EC show greater variability than that measured by CMR (Wood et al., 2014). For patients planning to receive device therapy or cardiac surgery with low LVEF, the variability in the assessed LVEF between modalities should be considered, and in this circumstances, in addition to LVEF cut-offs, other vital factors need to be worked up for decision making.

## Data Availability Statement

All datasets generated for this study are included in the article/supplementary material.

## Ethics Statement

This study is a retrospective study, all the data were obtained from PACS system, no patient personal information was revealed and this study did not have influence on patients’ diagnosis and therapy. All patients were diagnosed and treated with standard therapy in accordance with current guidelines. This study protocol and related details are fully reviewed and evaluated by Beijing Anzhen Hospital Ethics Committee. This study had ethics committee approval, the patient informed consent is waived.

## Author Contributions

LZ wrote the manuscript. AL and JT analyzed the data. LZ, AL, XM, JT, and JH revised the manuscript. All authors contributed to the manuscript and approved the submitted version.

## Conflict of Interest

The authors declare that the research was conducted in the absence of any commercial or financial relationships that could be construed as a potential conflict of interest.
